# Finding Purpose in the Conservation of Biodiversity by the Commingling of Science and Ethics

**DOI:** 10.3390/ani11030837

**Published:** 2021-03-16

**Authors:** John A. Vucetich, Ewan A. Macdonald, Dawn Burnham, Jeremy T. Bruskotter, Dominic D. P. Johnson, David W. Macdonald

**Affiliations:** 1 College of Forest Resources and Environmental Sciences, Michigan Technological University, Houghton, MI 49931, USA; 2 Wildlife Conservation Research Unit, Department of Zoology, Recanati-Kaplan Centre, University of Oxford, Oxfordshire OX13 5QL, UK; ewan.macdonald@sbs.ox.ac.uk (E.A.M.); dawn.burnham@zoo.ox.ac.uk (D.B.); david.macdonald@zoo.ox.ac.uk (D.W.M.); 3 Saïd Business School, University of Oxford, Oxford OX1 1HP, UK; 4 Terrestrial Wildlife Ecology Lab, School Environment and Natural Resources, The Ohio State University, Columbus, OH 43210, USA; bruskotter.9@osu.edu; 5 Department of Politics and International Relations, University of Oxford, Oxford OX1 3UQ, UK; dominic.johnson@politics.ox.ac.uk

**Keywords:** conservation psychology, governance, purpose, sustainability, values, virtue ethics

## Abstract

**Simple Summary:**

The biodiversity crisis, involving declines, even extinction, of many species, threatens the well-being and livelihoods of many people directly, and everybody indirectly, through a combination of its impacts on the functioning of ecosystems, availability of natural resources, and human values. Although conservation science itself is a trans-disciplinary blend of natural and social sciences, crucially it offers technical solutions to avert this crisis, thereby shedding light on the question of “what can we do?”; those answers inevitably raise the question “what ought we do?” and, again crucially, the answers must be sought in ethics. In this paper, therefore, we attempt the holistic commingling of sciences and ethics that is essential for individuals and societies to decide what to do about the biodiversity crisis. We identify several alternative ways forward, because there are several different ethical frameworks to guide the judgments that lie between evidence and action. Two of these are more familiar, deontology and consequentialism, whereas a third, virtue ethics, less familiar to many, might have great contemporary relevance. We explain all three, and show how each can guide modern citizens to a framework for thinking, without which a societal solution to the biodiversity crisis—ultimately the biggest crisis facing humanity—is impossible.

**Abstract:**

Averting the biodiversity crisis requires closing a gap between how humans tend to behave, individually and collectively, and how we ought to behave—“ought to” in the sense of behaviors required to avert the biodiversity crisis. Closing that gap requires synthesizing insight from ethics with insights from social and behavioral sciences. This article contributes to that synthesis, which presents in several provocative hypotheses: (i) Lessening the biodiversity crisis requires promoting pro-conservation behavior among humans. Doing so requires better scientific understanding of how one’s sense of purpose in life affects conservation-relevant behaviors. Psychology and virtue-focused ethics indicate that behavior is importantly influenced by one’s purpose. However, conservation psychology has neglected inquiries on (a) the influence of one’s purpose (both the content and strength of one’s purpose) on conservation-related behaviors and (b) how to foster pro-conservation purposes; (ii) lessening the biodiversity crisis requires governance—the regulation of behavior by governments, markets or other organization through various means, including laws, norms, and power—to explicitly take conservation as one of its fundamental purposes and to do so across scales of human behaviors, from local communities to nations and corporations; (iii) lessening the biodiversity crisis requires intervention via governance to nudge human behavior in line with the purpose of conservation without undue infringement on other basic values. Aligning human behavior with conservation is inhibited by the underlying purpose of conservation being underspecified. Adequate specification of conservation’s purpose will require additional interdisciplinary research involving insights from ethics, social and behavioral sciences, and conservation biology.

## 1. Introduction

Of the approximately 40,000 species of vertebrates known to inhabit the planet, approximately 20% are believed to be threatened with extinction [1]. Furthermore, human enterprises are believed to have increased the rate of species extinction by three orders of magnitude [2]. Circumstances are also grim for many of the species that will escape total extinction. For example, among the studied species of terrestrial mammals, the average species has been extirpated from two-thirds of its former geographic range [3]. Consequently, large portions of the earth’s terrestrial surface have lost more than half of their native species in historic times. Those losses represent a threat to ecosystem health. Causes of these losses include: habitat destruction and degradation (e.g., pollution), over-exploitation, introduction of non-native species, and deterioration of basic ecological relationships, especially predator–prey relationships [4,5,6].

Consider just the class of animals to which humans belong, Mammalia, over a fifth of which are currently threatened, a tally which might rise to 36% if those classified as data deficient turn out to be threatened [1]. As reviewed in Macdonald [7], of extant mammals, perissodactyles, primates, and elephants are the most threatened, with 75% of 16 species, 61% of 435 species, and 100% of two species listed as threatened, respectively. About 60% of the 74 largest terrestrial herbivores are threatened [8]. Of just one especially charismatic order, the Carnivora, about one quarter (26.4%, 78 species) are threatened, including 61% of the 31 largest species [9]. Drilling down further to just one family, the Felidae, at least 26 of 45 species are threatened or nearly so, and Sandom et al. [10] paint vividly the picture of how their disappearing prey, largely due to people, pulls the ecological rug from beneath their feet. Anyone doubting either the gravity of the extinction crisis, or the relevance to it of how people consider they ought to behave to nature, need look no further. So much for generalisations. Understanding often lurks in the detailed behaviour of individual non-humans [11] or the attitudes of individual humans (e.g., [12] and it is at that intersection that the purpose of conservation resides.

While the biodiversity crisis is very much about the relationship between humans and other animals, stemming the biodiversity crisis requires knowledge and insight from beyond the fields of zoology and animal ecology. As such biodiversity conservation has long been recognized as a transdisciplinary endeavor that includes increasing attention on the human dimensions of conservation—which includes social and behavioral science, politics, policy, economics, and ethics. Unbridged disciplinary divides exist not only between ecology and the human dimensions, but also within the human dimensions of conservation. The deepest divide in academia—between the sciences and the humanities—is represented in conservation by the chasm between conservation ethics [13] and conservation psychology [14]. This divide is easy to overlook because both disciplines share a basic interest in human behavior. This commentary addresses the challenges of synthesizing domains of knowledge from conservation ethics and psychology in efforts to address conservation problems.

## 2. Synthesizing Two Disciplines

Ethics is the academic field that aims to understand how humans ought to behave. The social and behavioral sciences aim to understand how humans actually behave and why. The distinction is sometimes represented informally by ethics’ concern with “ought” and sciences’ concern for “is” [15]. Conservation ethics, more precisely, aims to understand how we *ought* to relate to nature, who in the non-human world *ought* to be treated with moral consideration [16], and what the goal of conservation *ought* to be [17,18]. When the social and behavioral sciences are applied to conservation the focus is often on understanding why some people exhibit more pro-conservation behaviors than others [19].

Those disciplinary distinctions indicate a cardinal challenge of conservation. That is, to close the gap between is and ought. Drawing sharp attention to the gap between is and ought is salient because the importance and difficulty of minding that gap is routinely overlooked in conservation [20]. Some attempt to close the gap by disputing the distinction between is and ought and endeavoring to demonstrate that ethics can be subsumed by science [21]. Here, we offer an alternative approach to synthesizing principles from conservation ethics and conservation science—especially the behavioral and social sciences. The framework for this synthesis is governance by which we mean the regulation of behavior (of individuals and groups) by governments, markets, or other organization through various means, including laws, norms, and power [22]. Governance can include public policy and we use the term broadly enough for it to encompass, for example, management of a corporation or large NGO.

An important line of inquiry focuses on technical means of governing for the promotion of some social good, such as conservation (e.g., [23]). The line of inquiry we raise here stands beside that concern. Governance is inescapably an integration of empirical and normative elements. A simple example illustrates: Driving faster leads to more road deaths (empirical claim); we should set speed limits to 100 km/h (normative claim); driving faster than 100 km/h will result in a fine of some amount of money (governance). That example, beguilingly, skips past a venerable challenge of governance—that is, *how to appropriately* integrate the normative and empirical dimensions of governance [24]. The challenge is as old as governance itself. The challenge is also nearly intractable when expressed in such broad terms. A critical strategy for meeting this challenge is to narrow the scope. We narrow the scope by focusing on how ethics and (behavioral) science might be synthesized to guide governance as it pertains to conservation. One result of doing so is to reveal how conservation is impaired by an underdetermined and unresolved sense for the basic purpose of conservation. For emphasis and perhaps surprisingly to those less familiar with conservation, there are deep divisions within the community of conservation professionals about the underlying purpose of conservation [25]. Before tending that concern, we build a framework for synthesis by reviewing salient principles of ethics and behavioral science.

## 3. Ethics, Virtue, and Purpose

Academic ethics is comprised of three major frameworks: utilitarianism, deontology, and virtue ethics (Appendix A). Utilitarianism emphasizes that the rightness of an action is judged by the goodness of the consequences of the action, in particular actions with the greatest utility. Deontology emphasizes that the rightness of an action is judged by adherence to rules that stem from understanding duties and obligations owed to others. By contrast, virtue ethics emphasizes judgments about how a person is motivated to practice certain virtues and how well they practice those virtues. Below, we will focus on virtue ethics because, as we will see, it has deep connection to some basic principles of psychology. Interest in virtue ethics by applied ethicists has also been rejuvenated in recent decades—after centuries of relative neglect [26]. Being the least explored framework in modern times, virtue ethics may be fertile ground for conservation.

The roots of virtue ethics trace to Aristotle, who wrote that the purpose of a human life was to realize eudemonia—a word that while not readily translated into English, refers to a kind of human flourishing, a rich sense of happiness that transcends hedonism. Aristotle also believed this purpose in life would be realized in becoming increasingly proficient (wise) in the practice of certain virtues. For Aristotle, these virtues are represented by the Golden Mean [27]. The idea is exemplified by the virtue of being thrifty—a balance between two vices, greediness and wastefulness.

Of particular importance is the basic architecture of virtue ethics, which include the identification of a purpose in life—by which we refer to both lived purposes of real people and aspirational purposes that might be proffered philosophically. Virtues that would aid in realizing that purpose are subsequently identified and practiced. The proficient practicing of those virtues brings about the behavior required to realize the purpose. The architecture may be expressed diagrammatically: *purpose(s) in life* → *virtues* → *behavior*.

This architecture is emphasized by MacIntyre [26], which also develops an influential historical account of virtue ethics. Succinctly, the account runs roughly this way: Analysis of Homeric literature suggests that an important purpose in life was to become a heroic warrior. Accordingly, virtues that would serve such a purpose might include: bravery, cunningness, and perhaps even deceit. A different group, living in a different time or place, might acknowledge different purpose(s) and subsequently aspire to different virtues. According to MacIntyre [26], an important purpose in life for those living in ancient Greek city-states was to be a good citizen, suggesting the appropriateness of virtues, such as honesty, magnanimity, and forbearance. With the rise of Christianity and the intellectual contributions of St. Augustine, the purpose of life is taken to be moving from the “City of Man” to the “City of God”. The virtues most useful in realizing that purpose would include faith, hope, and charity.

With the dawn of The Enlightenment, God became an unsatisfying foundation for ethics. Immanuel Kant offered one of the first significant alternative footings for ethics (though he believed in God). Kant believed that ethics could be determined from pure reason alone. The next hundred years or so included numerous attempts to offer alternative footings. Hume believed that emotion and feeling, along with reason, is the foundation upon which to judge what is ethical. Kant and Hume revealed the shortcoming of each other’s view. Kierkegaard’s reaction was to claim that each human should be free to decide whether the foundation for judging right and wrong is to be reason or aesthetics (which corresponds very roughly to emotionality and egoism). Nietzsche took the failures of Hume and Kant to conclude that personal strength and will are the only sensible basis for deciding how to behave. Bentham and Mills believed the best foundation for ethics—in a world where the authority of God and monarch are second to the authority of human interests (secularism)—is to judge the rightness of an action by the goodness of the consequences of the action, in particular actions with the greatest utility.

While this account of virtue ethics emphasizes western culture, others have demonstrated commensurability between western notions of virtue ethics and ideas from Buddhism [28], Hinduism [29], and Confucianism [30].

An especially important feature of the preceding summary is its emphasis on the identification of purpose(s) in life which vary—for better or worse—among cultures living in different times and places. Yet, while purpose is fundamentally and inescapabilty normative, the development of a “good” purpose is not arbitrary insomuch as it should not be untethered from the most basic principles of reason and science—especially ecological science and behavioral sciences.

## 4. Behavioral Science and Values

**Figure 1:**
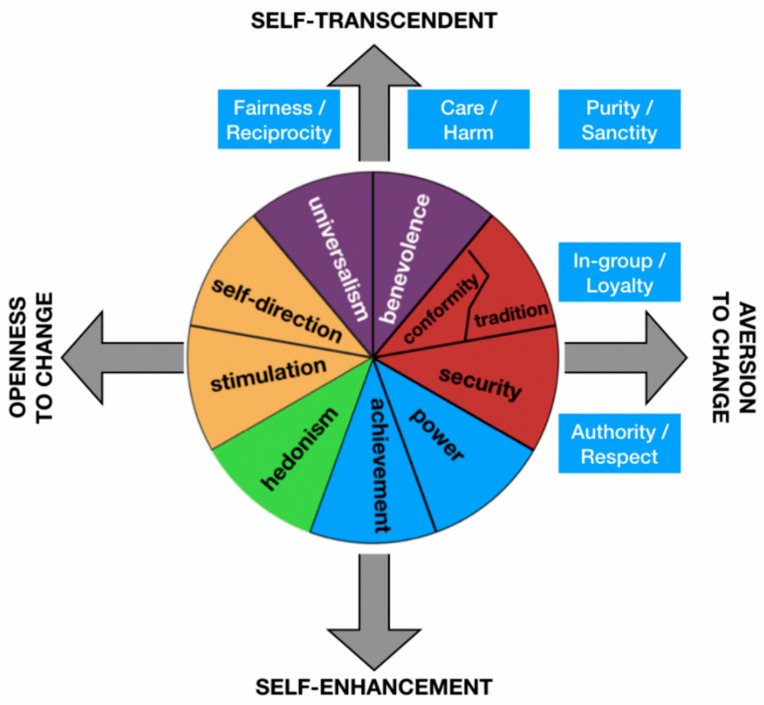
The theory of basic human values (TBHV) is a two-tier taxonomy, represented by the circle of 10 values arranged along two axes (transcendent–enhancement and openness–aversion). The values in this theory can be mapped onto the values of other theories, such as the moral foundations theory (MFT), which is represented by the blue boxes, which are located to indicated how the value categories of MFT correspond to the transcendent and aversion ends of the two poles in TBHV. See text for details. Adapted from Boer and Fischer [44].

Virtue is commensurate with psychologists’ notion of value (Appendix B), or what some psychologists refer to as transcendental values (TVs) to distinguish the concept from other concepts that use the word “value”, such as economic value or intrinsic value [31]. An important research program in psychology has been the identification of specific TVs and the prediction of behaviors from those values. A consistent finding of this research is that TVs are relatively few in number and appear to be held in common across diverse human cultures [32,33,34,35]. An important and representative taxonomy of TVs is the theory of basic human values (TBHV) [36], which includes 10 values oriented along two primary axes (Figure 1).

The usefulness of this taxonomy has been demonstrated in studies of more than 75 different cultures using two well-developed survey instruments [36,37]. While the taxonomies of particular research programs differ, they typically share important similarities and are sometimes viewed as one taxonomy being mappable onto another. For example, the two primary axes of Schwartz’s taxonomy correspond to the five dimensions of moral foundations theory (MFT, [33], Figure 1).

Within the taxonomy of TBHVs (Figure 1), one might think the challenge for conservation is to figure out how to encourage humans to better manifest values that are self-transcendent, like caring, and avoid values of self-enhancement, like power. Indeed, psychological research shows that power in this taxonomy tends to be negatively associated with pro-environmental attitudes [38]: Power seems to underlie many conservation problems and caring more could lead to improvements in conservation. Yet, some humans who actively oppose, let us say, the conservation of carnivores may still be especially caring (toward their family and friends). Similarly, some leaders of environmental NGOs are very power-hungry, yet do much for endangered habitats and species. In other words, any particular virtue can—often enough—serve wildly different purposes.

**Figure 2:**
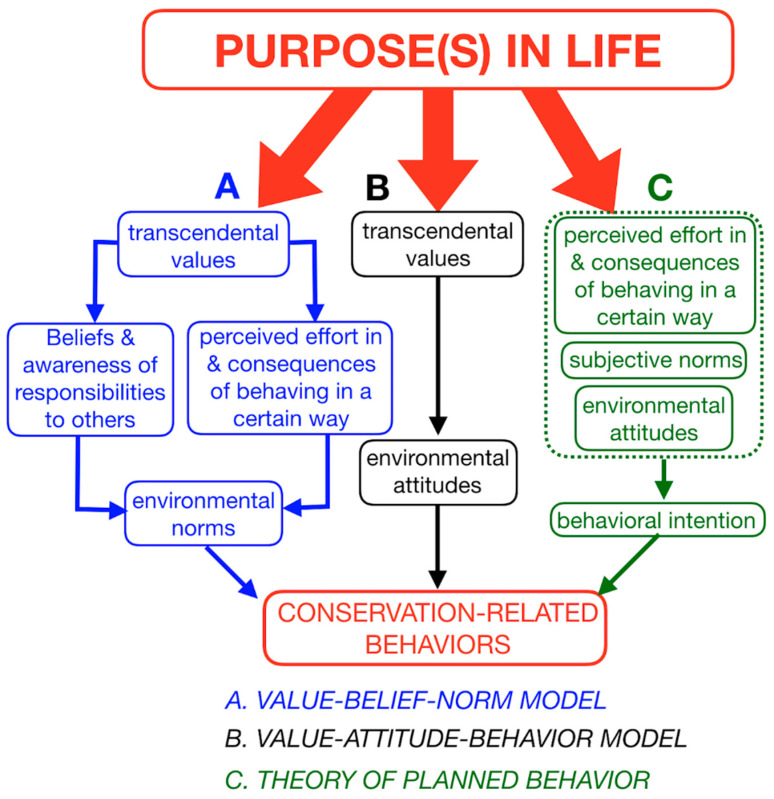
Some models used in conservation psychology to understand behavior. Many, though not all, such efforts make explicit use of transcendental values (or means-oriented values, Figure 1). Transcendental value is commensurable with the concept, virtue, as used in academic ethics. Model (A) can be depicted with additional mediating steps, i.e., *values* → *value orientations* → *attitudes & norms* → *behavioral intentions* → *behaviors*. The explanatory power of models in conservation psychology may well be extended by taking account of the purpose(s) in life to which one ascribes. In other words, ends-oriented values may be more important than generally appreciated. The important of purpose is highlighted by virtue ethics (see Section 3). For a more detailed review of these models, see Klöckner [45].

Among conservation psychologists, the effort to predict behaviors from TVs has been framed by efforts to account for processes that mediate the relationship between TVs and behaviors (Figure 2). One important way to conceptualize these mediating processes is through a hierarchy of cognition, whereby transcendental values are more basic (i.e., more general and more stable over time), and other types of cognitions (e.g., attitudes, intentions) are more context specific and malleable ([39,40,41], Model B in Figure 2). The basic insight to emerge from these models of behavior is that subjects who tend to express self-transcendence values (see Figure 1) also tend to exhibit positive attitudes about the environment and those tending to express self-enhancement (power) values tend to express negative attitudes about the environment [42,43]. These associations are not universal nor do they tend to be especially strong [44,45].

The tendency to not find strong associations amongst TVs, environmental attitudes, and environmentally relevant behaviors is thought to be explained, in part, by the importance and diversity of mediating factors. For example, values tend to be better reflected in behaviors when the value is *salient* (or activated in memory) at the time of the behavior and understood to be relevant to the behavior in question [46]. To illustrate, a person is more likely to choose an environmentally friendly product (over a less environmentally friendly product, but otherwise comparable) if they had been thinking about environmental issues just prior to the purchase and informed about how the purchase might affect the environment [47]. Activation of a value can also lead to a person seeking out relevant information that would influence a decision [48].

Other factors that might mediate the connection between behavior and TV are circumstances that make it difficult to exhibit pro-environmental behavior. For example, one might have a predilection to exhibit pro-conservation behavior but believe that doing so would not actually result in a benefit to conservation. To illustrate, one might appreciate bicycling to work rather than driving but feel that it will not benefit conservation so long as too few adopt that behavior. Such mediating influences are represented, for example, in the values–beliefs–norms model [49] and the theory of planned behavior ([50]; Figure 2). Another example of this kind of constraint involves the organization of societies that, for example, make it difficult to recycle even though such behavior is valued [51]. Other possible preventative circumstances are far more basic, such as economic insecurity ([52], but see [44]), or the perceived risks of exhibiting pro-environmental behavior, such as the risk of losing livestock (an economic harm) to carnivores [53].

## 5. Purpose Is Understudied in Conservation Psychology

Several lines of psychological inquiry posit that human behavior is importantly rooted to purpose (for reviews, see [54,55,56,57,58]). The psychological notion of purpose is importantly commensurable with the notion of purpose used in virtue ethics: purpose is more transcendent and abstract than means-oriented values and virtues (which are more specific and often in service of a purpose), and purpose is characterized by its content, strength, and one’s awareness of their purpose.

Most psychological research on purpose has focused on understanding the causes and consequences of the strength and awareness of one’s purpose(s). People with a strong sense of purpose tend to be happier, healthier, and wealthier [59,60,61,62]. A sense of purpose is also stronger among conservatives (compared with liberals; [63]), among those with a stronger sense of religiosity ([64], but see [65]), and among those who practice particular forms of meditation [66,67]. These and other patterns of this ilk evoke hypotheses about the causal mechanisms that lead to a stronger sense of purpose. The connection between strength of purpose and pro-conservation behaviors is essentially unstudied.

The content of individuals’ purpose(s) is also very much understudied. This circumstance may be attributable to thinking the range of purposes that could exist is strongly regulated by an individual’s culture; consequently, studying the content of purpose and passing normative judgment on the content of one’s purpose is of “minimal utility” for psychological inquiry [55,68]. The robustness of that explanation of the circumstance is far less important than the circumstance itself, i.e., the content of individuals’ purpose is not well studied in psychology—especially as the subject may pertain to understanding why individuals vary in the expression of pro-conservation behaviors.

The unaddressed questions are stark: Does the propensity to exhibit pro-conservation behavior vary with the strength or content of one’s sense for the purpose of their life? Is conservation fundamentally limited by the purposes that most people pursue? Is it possible to appreciably shape the content of life’s purpose of individuals or groups to favor conservation?

## 6. Synthesis

**Figure 3:**
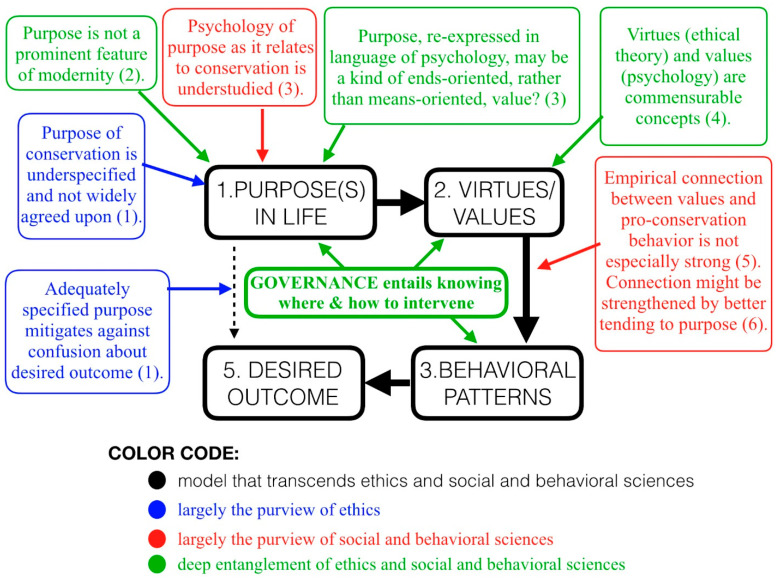
The black portions represent a simple model of behavior that transcends ethics and psychology. This model suppresses some of the intermediary steps depicted in Figure 2 to help draw attention to other more salient elements of the model. The nature of governance is represented from within that model of behavior (green). Colored portions (blue, green, red) from outside the black model imply with useful precision *how* to appropriately juxtapose the normative and empirical elements of governance as it pertains to conservation. Numbers in parentheses indicate sections of this paper that address the various boxed ideas: (1) Purpose in Conservation, (2) Purpose Lost, Purpose Found, (3) Purpose Is Understudied In Conservation Psychology, (4) Appendix B, (5) Behavioral Science and Values and (6) Figure 2. Values refers to “transcendent values” as that term is used in the main text.

The preceding is not a grab bag of ideas from disparate disciplines—juxtaposed for academic curiosity. Rather, we assemble these ideas to develop an argument for a unified purpose of conservation and governance. The first part of this argument is represented by Figure 3 and the second part begins to unfold in the next section (Section 7). The argument, if sufficiently robust, and promises a purpose that would foster pro-conservation behavior without neglecting concern for social justice. The argument implies the kind of governance that would promote, for example, protecting lions without neglecting the interests of humans who coexist with lions, conserving tropical forests without disregarding humans whose livelihoods have depended on using these forests, and allowing business the liberties to compete and flourish without risking the health of rivers, lakes, or oceans.

The black portions of Figure 3 depict a simplified model of human behavior that synthesizes insight from virtue ethics and behavioral science. Inside the black portion of Figure 3, one finds good governance represented as knowing where and how to intervene on behavior-related processes (and by implication when to refrain from intervening). Outside the black portions of Figure 3 are indications of how normative ethics and empirical science commingle in the service of conservation governance. Most importantly, we see indications that (i) the capacity to predict behavior may be advanced by empirical inquiry focused on the role that purpose plays in shaping behavior and (ii) the development of purpose (for an individual or group of individuals) entails a strong and inescapably normative element. These indications are more fully explored in subsequent sections.

The model in Figure 3 presumes that a role of governance includes, at least in principle, intervening on behavior of individuals and collectives for the purpose of promoting some normative aim. While the limits and hazards associated with such a presumption are significant and worth every moment of attention [69,70], the hazards of rejecting such a presumption are no less weighty [71,72,73].

## 7. Purpose Lost, Purpose Found

Conservation’s purpose—to avert the biodiversity crisis—seems straightforward and thoroughly unobjectionable (see Section 1). However, critical insight about the purpose of conservation may be found in what appears to be early stages of transformation for the purpose of economics and political theory. While all three disciplines—economics, political theory, and conservation—are especially important to human well-being, economics and political theory have had more influence on governance for a longer period of time. We posit, if conservation is to play a substantive role in the future well-being of humans, then conservation’s purpose may also require transformation like that which may be occurring in economics and political theory.

We follow Raworth [74] (pp. 32–40) in recounting transformations in the purpose of economics: When the word was invented more than two millennia ago, economics referred to the art of household management and set distinct from another then newly-coined word, chrematistics, the art of acquiring wealth. In the 1760s, as modern science was being born, James Stuart defined “political economy” as a science whose explicit normative purpose was to guide domestic policy for free nations aiming to secure living and jobs for all in a mutually thriving community. A decade later, Adam Smith articulated a similar normative purpose for the science of economics. Its first purpose is to enable citizens to provide themselves with “plentiful revenue”. Its second purpose is to enable nations to supply themselves with “revenue sufficient for the public services”.

Two generations later, John Stuart Mill defined political economy as a science which “traces the laws of such of the phenomena of society as arise from the combined operations of mankind for the production of wealth”. The emphasis was describing laws. That is, describing how the world “is”. Attention and detail that had been given to the normative purpose of describing those laws was quietly reduced to very simply the “production of wealth”.

Three generations later, in 1932, Lionel Robbins completed the dissolution of normative purpose in political economics by defining it as a “science which studies human behaviors as a relationship between ends and scarce means which have alternative uses”. That is, economics is a science whose purview is to understand “what is” without regard for “what ought to be”. This conceptualization, purportedly value-neutral, dominated the minds of the most influential economists for much of the 20th century—especially the Chicago school of economics.

In truth, this conceptualization not only obscured powerful (and questionable) ethical presumptions [75,76], it also created a purpose vacuum that was filled by the mid-20th century; that is, efficient, perpetual exponential growth of nations’ gross domestic products. Perpetual GDP growth landed in the seat of purpose not because of any reflected upon normative reasoning. Rather, it resulted from a newly acquired capacity to measure GDP and heat-of-the-moment political reactions to economic crises of the interwar period.

This centuries-long transmogrification of purpose in economics is consistent with MacIntyre [26], who also makes a case that modernity brought a broad cultural shift in the perception of life’s purpose—a shift that persists to the present day. Purpose, so it is argued, was reduced, in a word, to efficiency. That is, efficiency for its own sake. Efficiency aimed in any direction. The case is a simple corollary of the theory of bureaucracy developed by Max Weber (a founder of modern sociology), which explains how Western society elevates the role of efficiency over other normative purposes in the 20th century technocratic life [77].

If modernity witnessed the dissolution (and reconstitution) of economics’ normative purpose, then the Anthropocene—with its attending crises of the environment and social justice—will almost certainly usher new developments in purpose. Early signs suggest that a new normative purpose for economics may be something like the realization of “human prosperity [broadly construed] in a flourishing web of life”, where web of life includes various manifestations of human and non-human life [74]. Impressive signs of economic’s changing purpose may be found, for example, in Stiglitz [78].

One may think, at this point: yes, good for economics. Conservation has always had an explicitly and broadly benevolent purpose—avert the biodiversity crisis. Unfortunately, the case may not be quite that simple. Is averting the biodiversity crisis the ultimate purpose of conservation? Or is it a proximate purpose, with the ultimate purpose of conservation to serve only the well-being of humans? Or is the purpose of conservation to serve the well-being of humans and nonhuman life—especially species, populations, and ecosystems?

Before exploring these wrinkles in conservation’s purpose, allow us to briefly review how social justice and political theory are also experiencing their own developments in purpose, summarized here from an account detailed in Nussbaum [79]. These disciplines rose from an explicit presumption about what the purpose of life ought to be. From Thomas Hobbes (17th century philosophy, of “nature red in tooth and claw” fame) to John Rawls (one of the most influential political theorists of the 20th century), political theory has largely presumed that the purpose of life ought to be: cooperation among approximate equals in search of mutual benefit in their public lives [79,80]. This foundation ultimately fosters real-world biases based on gender, race, wealth, and health that grow into profound injustices observable within households and nations and between neighborhoods and nations. This understanding of purpose (associated with contractarian understandings of political theory) spurred a more recent and countervailing view associated with the, so-called, capabilities approach to social justice [81]. This approach to social justice presumes that the purpose of a human life is to cooperate for the purpose of allocating entitlements among those to whom entitlements are owed, including humans and non-humans (Appendix C). This understanding of social justice has gained substantive traction, for example, in the activities of the United Nations, not least their Sustainable Development Goals.

As modernity gave way to the Anthropocene, economics and social justice—domains of scholarship with profound influence on well-being—have begun to show signs of developing a richer, more robust account of normative purpose. If conservation is to be taken by the world’s leaders with similar gravitas, conservation leaders may likewise be wise to reach for a deeper understanding of conservation’s purpose and its relationship to other domains of life.

## 8. Conservation

### 8.1. Purpose of Conservation

Conservation would seem to have an abundantly clear purpose: avert the biodiversity crisis (see Section 1). Conservation’s more proximate purpose entails means by which to do so, such as eliminate over-exploitation of species and restore the health of ecosystems upon which species depend. Useful as these expressions of purpose are, they have at least two critical limitations.

The first is highlighted by asking, *why* do we wish to avert the biodiversity crisis? Is it predominantly because human well-being depends on averting the crisis? Or is the well-being of non-human life—beyond its value to humans—also a critically important reason for conservation? In other words, is conservation an anthropocentric or non-anthropocentric endeavor? If the latter, non-anthropocentric in precisely what manner? Those questions run like fault lines through the conservation community. The answers say much about the purpose of conservation, because many aspects of biodiversity are not sufficiently or obviously important to human well-being.

A second limitation to conservation’s unembellished purpose—i.e., avert the biodiversity crisis—is highlighted by asking how does this purpose relate to other basic public interests, especially economic freedoms, personal liberties, social inequalities, and obligations to treat individual animals with concern for their wellbeing. The precise meaning of these interests and the importance placed on them—in relationship to conservation—influence greatly answers to questions like: under what conditions is it acceptable to kill animals in the name of conservation and, under what conditions is it acceptable to infringe on the liberties and well-being of some humans in the name of conservation? Two specific cases, of many, are trophy hunting lions to conserve lion habitat [20] and displacing destitute refuges to protect endangered elephants [82]. That these public interests—human well-being, animal welfare, and biodiversity—interact in such a basic way indicates the need for a purpose of conservation that is deeply and mindfully integrated with other social purposes.

The community of conservation professionals may represent perhaps four distinct purposes for conservation. *New conservation* emphasizes averting the biodiversity crisis primarily for the ultimate purpose of benefiting human wellbeing [17]. What might be called *orthodox conservation* emphasizes that species and ecosystems possess intrinsic value (Appendix D) and averting the biodiversity crisis is its own ultimate purpose—aside from whether doing so satisfies human wellbeing. This perspective is represented by, for example [83], Soulé [84], and the mission statement of the Society for Conservation Biology [85]. *Compassionate conservation* may be seen as a reaction to the increasing use of killing as a conservation tool, which in turn is a reaction to the widespread impact of non-native and invasive species. It believes that conservation cannot fulfill its purpose when it relies as often as it does on killing individual animals [86]. Another vision of conservation sees a close association between its purpose as maintaining and protecting hunting heritage [87,88]. While this vision is rooted to North America, the connection between hunting and conservation has an important influence in, for example, Europe [89] and Africa [90]. Other fault lines through conservation’s purpose are indicated by perspectives offered in Treves et al. [91], Washington et al. [92], and Vucetich et al. [93].

Realizing a well-conserved world likely depends on an adequately specified purpose that is widely supported by the conservation community and subsequently embraced by society at large. Neither circumstance has, thus far, been realized. We are not arguing for universal agreement to the *n*th degree; however, there is almost certainly a need for broader agreement on a more richly specified purpose of conservation.

### 8.2. Purpose(s) in Life

The preceding pertains to conservation’s purpose at an institutional level. This *institutional purpose*—avert the biodiversity crisis, for whatever ultimate reason and by whatever acceptable means—would not be a *personal purpose* of life for the vast majority of individual humans. Yet, there is a need to better understand what personal purposes are most capable of advancing conservation.

Conservation professionals recognize two broad motivations for conservation that are relevant for the consideration personal purposes. One motivation is anthropocentric and the other is non-anthropocentric. Conservation professionals are not of like mind about which motivation is appropriate [25]. Prior work [18] suggests that an anthropocentric vision of conservation is consistent with a personal purpose of life, something like:

Consume products, material and energy as much as desired without infringing on interests of other humans (present and future) to do the same.

A non-anthropocentric vision of conservation is, however, consistent with a personal purpose, something like:

Consume products, material and energy as little as necessary to maintain a healthy, meaningful life.

The phrase “healthy, meaningful life” is less vague and subjective than might be presupposed. This concept is, for example, subject to considerable objective reasoning through social and behavioral sciences [93]. Yet, its meaning is also subject to normative considerations about, for example, fair allocations of resources [94]. This latter non-anthropocentric purpose is also flexible enough to provide corrective guidance to those using more than appropriate and to those using less than is fair.

Those two purposes would lead to very different worlds. Moreover, one might not be surprised if psychological inquiry were to indicate that increased affinity for the first anthropocentric purpose is associated with behaviors that exacerbate the biodiversity crisis. If so, it would likely be due to the first purpose’s emphasis on consuming as much as desired [95].

The second non-anthropocentric purpose, however, seems well aligned with emerging purposes in economics and social justice. The second purpose may also reduce risks of unintended consequences of well-intended policy [95], including cases where well-intended (or well-purposed) policies are manipulated by citizens who do not share the motivating purpose. One example of such manipulation is when the US policy for biofuel (a possibly well-intended policy) led to overexploitation of Indonesia’s forests [96]. A more general example is Jevon’s paradox, whereby well-intended gains in technological efficiency do not result in reduced consumption. For example, increased fuel efficiency of US automobiles in past decades was followed by increased rates of driving, not reduced fuel consumption [97]. Citizens that share a genuinely pro-conservation purpose are more likely to follow the spirit of pro-conservation policies, rather than merely adhere to such policy in a technical manner.

Finally, the aforementioned personal purposes would undoubtedly interact with other important and more varied personal purposes, ranging from being an entrepreneur, family provider, artist, teacher, patriot, child of God, and myriad more. Far too little is known about which of the most common purposes in life align well with conservation’s purpose or pro-conservation behaviors that would rise from those purposes.

### 8.3. Governance

Allow us to suppose, for the sake of illustrating a point, that “*consume as little as necessary to maintain a healthy, meaningful life*” is the purpose most capable of averting the biodiversity crisis. If the biodiversity crisis ranks among humanity’s most basic challenges, then to what extent ought that purpose—or any such purpose—be inherited by government? Intriguing as the question may seem, it covers too much ground in a single step. Allow us to begin by noting that the biodiversity crisis is related to a phenomenon that economists classify as overexploitation of a common-pool resource (CPR).

### 8.4. Common-Pool Resources

Considerable research indicates that CPRs can be sustainably exploited by small groups of self-governed humans under a set of eight conditions [98]. One condition is “well-defined boundaries around a community of users” [99,100]. That boundary includes a sufficiently shared sense of purpose (or end goal) about the resource and its exploitation [23]. This sense of purpose would be expressed through attitudes and norms of behavior, where deviators are admonished lightly and in graduated fashion and adherents are conspicuously rewarded [23].

Two other conditions (traditionally denoted as conditions 7 and 8) stipulate that the small group of self-governed are embedded in a larger network of governance (e.g., regional and federal government) that fosters (or at least does not interfere with) the group’s purpose.

If the three conditions hold—along with the other five conditions developed by Ostrom [98] and further evaluated by Cox et al. [99]—then self-governance by a small group may outperform resource management dominated by top-down regulation or privatization (each of which have their own demanding conditions for realizing sustainable exploitation).

Humans do not necessarily adopt those eight conditions for sustainable exploitation automatically. Additionally, developing a shared purpose in a small group bound only by a CPR can be challenging, but not dreamy. However, as the group becomes larger and more diverse, so too increases the difficulty of developing a sufficiently shared purpose [23]. In summary, principles of governance acknowledge (if not underplay) the central importance of shared purpose.

### 8.5. National Governments

National governments have purposes that evolve and develop over time. For example, several nations of western Europe took as an important purpose the allocation of welfare in the years following World War II. During the last two decades of the 20th century, the US and UK governments stepped away from that purpose, arguably toward the purpose of promoting principles of free-market economies (sensu [101,102,103]).

Citizens embrace national purposes to varying degrees (including explicit opposition). However, there is always sufficient support (or insufficient opposition), on the whole, to sustain the purpose for as long as it persists. Developments in a national government’s purpose are sometimes intentionally driven and sometimes result in unintended consequences.

Given the preceding, to what extent—if at all—should a national government embrace a purpose akin to “*consume as little as necessary to maintain a healthy, meaningful life*”? A key consideration would seem to include a nation’s proclivity for economic and personal liberty. In more liberal nations, any such purpose would root (or wither) via a “free” market of ideas. In less liberal nations, any such purpose would depend more on marketing a purpose-idea to government officials. In either case, the fate of a purpose-idea would seem to benefit from understanding the supportive roles of robust reason [104,105], marketing and campaigning [106,107], the sociology and history of social change, and how policy-relevant ideas spread [108,109,110].

Embracing a non-anthropocentric purpose of conservation in more liberal societies likely requires understanding any such purpose in at least three particular ways:

1. As an ecologically informed understanding of the law of equal liberty (i.e., freedom up to the point of undue infringement on others’ well-being), rather than a well-intended though overly exuberant imposition on liberty [71].
2. Not as an austerity program, but as the removal of obstacles to freely pursue a healthy meaningful life. The prospect for seeing the non-anthropocentric purpose in this way is positive, as indicated by a recent increase in governments’ interest in policies informed by the science of subjective well-being [111].
3. As being about “happiness” *now* and thus in tune with humans’ innate tendency to discount the future [112,113] and quickly return to a relatively stable state of happiness following major negative or positive life changes [114].

We do not claim that establishing these understandings would be easy—but it is plausible that they are necessary, at least during a phase that would precede widespread adoption of the purpose.

Advancing a purpose like, “*consume as little as necessary to maintain a healthy, meaningful life*” is not fantastical. For example, Bhutan demonstrates that an entire nation can set personal well-being as a formal purpose of government. Some western leaders have developed proposals that point in the same direction, such as Stiglitz et al. [78], which was commissioned by French President Sarkozy.

Also relevant to the prospect of a nation’s embrace of this purpose is international power dynamics. Thus, it is important to ask, how international power dynamics would be affected—over shorter and longer time frames—by nations embracing or rejecting any such purpose to varying degrees.

Finally, the world’s top 100 economies include 31 nations and 69 corporations [115]. Advancing a pro-conservation purpose will depend as much on national governments as it will on corporations’ purposes and their sense of corporate responsibility [116].

These matters of governance are generally relegated to arcane discourse among political theorists and often the subject of acrimonious proclamations by political commentators. Yet, the analysis presented here suggests these matters of governance ought to be treated with great interest by conservation professionals of every stripe.

### 8.6. Ethical Knowledge

A purpose we have been considering—*consume as little as necessary to maintain a healthy, meaningful life*—emerges from considering a particular vision of non-anthropocentrism, which has been labelled just conservation [93]. An important topic for future scholarship would be whether other visions of non-anthropocentrism—especially just preservation [91] and ecojustice [92]—lead to purposes that would be appreciably different?

In any case, the frontier of non-anthropocentrism—where we believe one will find a richer understanding of conservation’s purpose—lies in knowing: (i) how to act in cases where resource scarcity precludes meeting all the interests of both humans and nonhumans and (ii) precisely what counts as resource scarcity. That is, when exactly is conservation a zero-sum game and when is it not? What counts as a win-win outcome? When are win-wins possible and when are they not? Those outstanding questions demand attention from ecosystem science (e.g., [117]) and behavioral science (see the next section), but they also deserve the scrutiny of conceptual analyses (e.g., [93,118]).

According to basic principles of social justice, a zero-sum game may be resolved fairly by giving due consideration to each of four principles: need, equality, equity, and entitlement [94,119]. The precise weight of consideration for each principle is the focus of discourse and insight in western political theory—ongoing for the past 2500 years. This discourse generally assumes that the two agents involved in the zero-sum game are both human. An outstanding question: How, if at all, do insights about need, equality, equity, and entitlement apply to zero-sum games where some of the agents are not human? This question is only beginning to be explored (Vucetich et al. [93] and references therein).

The centuries-old discourse on justice in the West is also rich with discussion about the relative weight given to individuals versus collectives (of humans)—from Plato’s *Republic* to Karl Popper’s *Open Society* (1945). The difficulty of this discourse hints at how much more complicated conservation is made by its purview to take account of an even broader set of concerns: individual humans, human collectives (NGOs, corporations, and nations), individual nonhuman animals, and ecological collectives (populations, species, and ecosystems).

The conservation literature gives the impression, often enough, that the biodiversity crisis is associated with giving too much emphasis to utilitarianism and insufficient emphasis to deontology (e.g., [120]) (those frameworks, deontology and utilitarianism, are defined in Section 3). Yet, ethical theory provides no reason to think those frameworks place a constraint on the inclusiveness of one’s moral community. As such, an outstanding question is: How are moral judgments pertaining to conservation informed by the sometimes-conflicting principles of deontology and utilitarianism?

These may be among the most important questions to pursue via humanities scholarship to better understand conservation’s purpose and its manifestation in the world.

### 8.7. Empirical Knowledge

Consider, as an example, just one issue from the preceding section—say, the relative weight given to equality and entitlement (Appendix C) when making moral judgments pertaining to conservation. The collective scholarship from the humanities on justice is importantly an assessment of the perils of leaning too hard on one principle or the other. Much insight on the same topic is also found in empirical scholarship. For example, the weight given to equality (as opposed to merit or entitlement) varies across cultures, with candidate explanatory variables, such as the society’s degree of Westernization, mode of production (industrial, hunter-gatherer, agriculture), and frequency of anonymous social interactions [121,122,123]. This single example is an exceedingly modest gesture toward a large body of empirical insight on the sociology and psychology of social justice (e.g., the journal, *Social Justice Research*).

Social psychology has long appreciated the feasibility and pitfalls of shaping behavior by intervening at various points in the causal chain that precedes behavior. For example, it is difficult to change TVs [69] and TVs may not be especially constraining on behavior. Much insight lies with empirical knowledge about the effectiveness and limits of intervening on the cognitions that precede behavior [124]. Behavior can also be favorably modified by structuring society in ways that make desired behaviors easy and undesired behaviors difficult [51]. Far less is known about the behavioral consequences of attempting to shape another’s *purpose*.

A society’s basic values (including its purposes) are manifest through various elements of a society in ways that are self-reinforcing [69]. Those forces lend stability to a social group, but they are an obstacle when seeking change. Intentionally advancing a pro-conservation purpose requires empirical knowledge about those reinforcing processes.

Finally, advancing a pro-conservation purpose would likely be aided by principles of:

(i) Natural governance—which is mindful of the ecological and evolutionary processes that shaped human behavior [24]—though the most effective form of such theory and its integration with real-world governance seem to be matters of debate (compare, for example, Pinker [125] with Wilson [126], Bellah [127], and Turchin and Gavrilets [128]) and;
(ii) Libertarian paternalism [129] and choice architecture—which aim to “nudge” people toward making decisions that favor certain behaviors without undue infringement on liberty [130].

### 8.8. Cultural Imperialism

Advocating for a pro-conservation purpose can be, undeniably, cultural imperialism. It would be so toward any culture who valued (in principle or in practice) the unsustainable use of natural resources, unbounded consumerism, economic growth, and gross inequality. The cultures that most comes to mind are some of the cultures sometimes described as WEIRD (Western, educated, industrial, rich, and democratic).

Furthermore, and still suppose that “*consume as little as necessary to maintain a healthy, meaningful life*” is the purpose most capable of advancing conservation: The notion of a “healthy, meaningful life” is flexible enough to honor cultural variation, yet the notion is not vacuous given the philosophical and scientific knowledge of subjective and objective well-being [114].

## 9. Conclusions

Conservation-related behaviors are explained by many factors (Figure 2). Among the important mediating influences may well be virtues and means-oriented values—like caring, loyalty, or any of the values that compose moral foundations theory (Figure 1). However, any means-oriented value may serve a wide range of purposes or ends-oriented values—whether that be a pro-conservation purpose or some purpose that works against conservation. As such, means-oriented values (and virtues) pose little constraint on one’s purpose. If a well-conserved Earth depends on understanding various relationships between purpose and behavior, then scholarly inquiry synthesizing these disciplines will be especially valuable.

Purpose also lies in the soul of virtue ethics and governance. Contemplating and manifesting one’s existential purpose are also what make us uniquely human. Purpose in economics and social justice has developed over time and may again be showing early signs of change. Those changes in purpose would be tectonic. Those nascent changes may also indicate the direction conservation’s purpose is liable to take—especially if conservation is to be counted by social leaders as an endeavor for the inclusive and collective welfare of the planet’s denizens. At present, the purpose of conservation is either underspecified (avert the biodiversity crisis, but to what end and by what means?) or contested.

The path to a rebooted purpose capable of advancing conservation is slow and full of pitfalls. Those circumstance do not lessen what appears to be the necessity of fostering such a purpose. The necessity is born from any interest to allow humans the liberty to pursue healthy, meaningful lives and coexist with each other and myriad forms of biodiversity—whales, mangroves, bees, peatlands, panthers, and the rest.

## Data Availability

Not applicable.

## Appendix A. Primer on Three Ethical Frameworks

To highlight distinctions (and similarities) among the frameworks, consider a heuristic example: Is it acceptable to squash a spider in the house, as opposed to escorting it outside? The utilitarian would emphasis the decision with the greatest utility. Before doing so, the utilitarian has to decide whose utility is of concern in the first place—only the human, or both the human and the spider. The deontologist would ask is the spider the kind of creature to which we have any obligations; if so, what are they. The virtuous person might be moved by feelings associated with the virtue of kindness—a virtue that for one reason or another has been selected for cultivation—and would likely escort the spider outside.

In many cases, the three ethical frameworks would endorse the same decision, but not always. While the three frameworks differ significantly in emphasis, they are not wholly independent of each other (e.g., [131]). Moreover, psychological research indicates that real human decisions routinely draw on principles from more than one ethical framework [132,133].

One might think—if only superficially—that conservation is justified as well by one framework as the next: Maintaining healthy populations of pollinators will result in the greatest utility to the greatest number. We have a duty to protect mangroves and coral reefs for future generations. The virtuous person would protect endangered rhinos.

Indifference over ethical frameworks is shortsighted for not recognizing that each framework has its own limitations. Utilitarianism does not work especially well when there are difficulties predicting or fully accounting for the consequences of an action. Deontology sometimes seems hamstrung by conflicting duties that are not easily adjudicated. Virtue ethics is challenged when there is insufficient insight to guide decisions about what virtues should be espoused and how they ought to be directed (more on this below). The limitations do not paralyze ethical decision-making. In some cases, they are caveats to be accommodated. The complimentary nature of these frameworks also suggests that the most robust conservation ethic is thoughtfully pluralistic with respect to these frameworks. This being said and for reasons given in the main text, we focus on virtue ethics.

## Appendix B. Virtues and Values—A Bridge

We assume readers have brought a colloquial understanding of the concept of virtue. To have done so is useful. Nevertheless, there is insight in reflecting on a more formal understanding. The ethicist’s notion of virtue is well represented by MacIntyre [26]:
Virtues are dispositions not only to act in particular ways, but also to feel in particular ways. To act virtuously is not, as Kant was later to think, to act against inclination; it is to act from inclination formed by the cultivation of the virtues.

A critical bridge—a point of commensurability—is built between ethics and behavioral science by recognizing that virtues are strongly comparable to values—as that concept is employed in a technical sense by many behavioral scientists. This notion of value in the discipline of psychology is well represented by Schwartz [134] ([37], p. 222):
Values are beliefs linked inextricably to affect (emotions)… Values refer to desirable goals that motivate action… Values transcend specific actions and situations… Values serve as standards. They guide the selection or evaluation of actions, policies, people, events, and people, including oneself… People decide what is good or bad, justified or illegitimate, and worth doing or avoiding on the basis of possible consequences for their cherished values…

Following Raymond and Kenter [31] and others, we refer to these as transcendental values (TVs). Doing so helps distinguish this concept from other distinct concepts that also happen to use the word “value”, such as economic value or intrinsic value.

Aside from the obvious similarities between values and virtues, we draw attention to similarities that might have been overlooked: the role of emotion in TVs is analogous to the role of “feeling” in virtues. Additionally, the dispositional nature of a virtue is analogous to the notion that TVs “transcend specific actions and situations.”

## Appendix C. Entitlement

The term “entitlement” is jargon, related to, but distinct from, important understandings of rights. For example, voting is a right for women in France that was *created* by a social contract in 1945. However, French women were entitled to vote before 1945. The existence of the entitlement motivated the creation of the right. Entitlement is also distinct from desert—noun form of deserve—in the sense that entitlements are not necessarily earned by merit.

## Appendix D. Non-Anthropocentrism

This appendix is a summary of Vucetich et al. [16] and a near-verbatim presentation of Appendix C in Vucetich et al. [20]. To be non-anthropocentric means to acknowledge that at least some elements of nonhuman life possess intrinsic value. An object possesses instrumental value if it is valuable as a means to some other end, and possesses intrinsic value if valuable beyond its instrumental value or valuable for its own sake [135]. If something possesses intrinsic value it means essentially that we have an obligation to treat it with respect or fairly and with at least some concern for its wellbeing or interests.

That individual vertebrate organisms possess intrinsic value is widely accepted. One of the more important lines of reasoning begins with the supposition that humans possess intrinsic value because we have interests (e.g., to avoid pain and to flourish). Given that supposition, it follows that any entity with such interests would also possess intrinsic value. Because all vertebrate organisms possess those interests, they also possess intrinsic value. The force and universality of that reasoning is indicated by the principle of ethical consistency, i.e., treat others as you would consent to be treated in the same position (for a general review, see [136]; for applications to nonhumans, see [137,138], p. 12 of [139]; see Nussbaum [79] for a similar application of a traditionally anthropocentric ethic to nonhumans). Most human cultures are undergirded by some variant of this principle (e.g., the golden rule). The intrinsic value of at least some non-human portions of nature is also widely appreciated—as reflected by sociological evidence [140] and the laws and policies of many governments.

Because all living things have an interest to flourish, a case has been made that all living things possess intrinsic value—a position known as biocentrism [141,142]. While the case for complete biocentrism may not be so widely appreciated, the case for intrinsic value of mammals and birds is essentially undisputed. That later case is more than enough to support claims we make about the purpose of conservation and sustainability.

While those considerations pertain to the intrinsic value of organisms, different reasoning is required to consider the intrinsic value of ecological collectives, such as populations, species, and ecosystems. One line of reasoning would be that ecological collectives are normally homeostatic, resilient, and interconnected and that those properties imbue them with intrinsic value [143]. Some, but not all, ecologists believe that ecological collectives are *not* characterized by those properties (e.g., [144,145]). Nevertheless, whether an ecological collective possesses those traits is not entirely a scientific question, but is in an important sense a metaphysical question (to illustrate: *Describing* the interconnectedness of a system is usefully considered a purely scientific endeavor (but see [146]); but *judging* whether those interconnections are sufficiently intimate for the system to qualify, for example, as an organism involves a significant metaphysical considerations (e.g., [147,148]). As such, it is at least partially relevant that many (if not most) people believe that “nature possesses a delicate balance that is easily upset by humans [149]”.

A second line of thinking (also developed by Leopold [143]) also supports the intrinsic value of ecological collectives. This line of thinking begins with the supposition that we humans, along with ecological collectives, are members of the same biotic community. In sharing community membership, and by extending the moral principles that apply to human communities, we ought to treat ecological collectives with respect.
